# Total Knee Arthroplasty in Ochronosis Arthropathy: A Case Report and Systematic Review

**DOI:** 10.1155/2019/1871856

**Published:** 2019-10-09

**Authors:** Wu Chean Lee, Tong Leng Tan, Ying Ho Chan

**Affiliations:** Department of Orthopaedic Surgery, Tan Tock Seng Hospital, 11 Jalan Tan Tock Seng, Singapore 308433

## Abstract

**Introduction:**

Ochronosis arthropathy (OcA) is a rare condition which may be treated with total knee arthroplasty (TKA) at the end stage. The condition is often discovered only intraoperatively and the ideal choice of TKA is unknown.

**Case Presentation:**

A 54-year-old male with worsening chronic bilateral mechanical knee pain had failed conservative therapy. Posterior stabilised (PS), cemented TKA and patella resurfacing was performed. Intraoperatively, collagenous structures such as the menisci and cartilage were noted to be black. Histological examination showed deposition of large amorphous brown material suggestive of ochronosis. He recovered well and underwent TKA of the contralateral knee the following year. At 2 years postindex TKA, his outcome scores improved and he was satisfied.

**Discussion and Conclusion:**

With increasing TKA performed worldwide, a surgeon may eventually be surprised by the above findings once in their lifetime. However, OcA may be considered a likely diagnosis and it is safe to proceed with TKA. There is no particular TKA design that proved to be superior in our systematic review of 19 publications regarding TKA as all reported good outcomes. However, as the pathogenesis of OcA appears to be inflammatory in nature, we suggest using cemented PS TKA with resurfacing of the patella.

## 1. Introduction

Ochronosis arthropathy is one of the manifestations in patients with alkaptonuria [1]. Alkaptonuria is a rare autosomal recessive disorder with an estimated prevalence ranging from 1 : 19,000 to 1 : 1,000,000 [1]. The disorder leads to a defective enzyme homogentisate 1,2-dioxygenase (HGD), resulting in accumulation of homogentisic acid [1]. They may deposit in collagen-rich connective tissues to form yellowish discoloration, hence the term ochronosis (ochre: yellow in Greek) [1]. These changes make the connective tissue more brittle and chemically irritate the joint, causing degeneration of the joint with eventual end-stage arthritis, with the knee being most commonly affected [1].

Total knee arthroplasty (TKA) has been suggested as an effective treatment for end-stage ochronosis arthropathy but these are limited to case reports [2–8]. A critical review of 13 case reports seemed to suggest no difference between cemented and cement-less TKA for ochronosis arthropathy [8]. However, other aspects of TKA such as cruciate substitution or retaining and patella resurfacing were not studied.

Although the condition is rare, the aging society and increasing volume of TKA will mean surgeons may eventually encounter this [9]. Owing to its rarity, the ideal choice of implant has not been elucidated. In this study, we present a case of ochronosis arthropathy treated with TKA to contribute to the literature. Secondly, through a systematic review, we aim to determine the appropriate choice of TKA implant for this condition.

## 2. Case Study

A 54-year-old male presented to us with bilateral mechanical knee pain with the right knee being worse. Functionally, he could not climb stairs and could only walk for about 5 minutes.

His significant medical history included diabetes mellitus, ischaemic stroke, cervical spondylosis, left frozen shoulder, right calcaneal tendon rupture which was repaired 14 years prior, and right lower limb peripheral vascular disease which was treated with angioplasty 1 month prior.

On physical examination, there was medial joint line tenderness. The range of motion (ROM) was 5° to 100° with palpable crepitus. The dorsalis pedis pulse was not palpable, but the posterior tibialis was palpable.

His preoperative Oxford knee score (OKS) [10] was 9, while his Knee Society (KS) function score [11] was 0. The KS knee score was unavailable.

**Figure 1:**
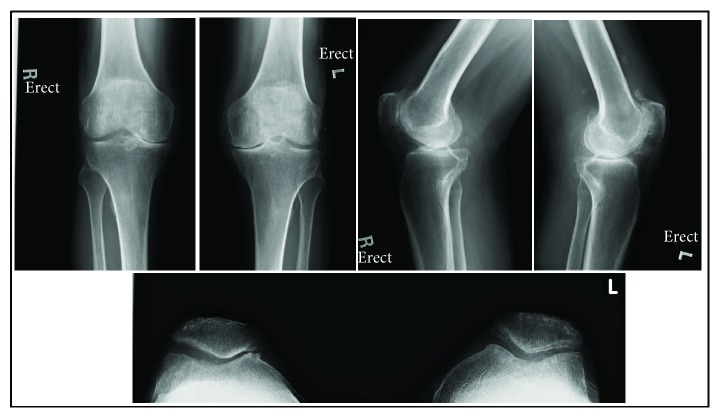
Pre-operative X-rays of the knees.

X-rays of the knees showed advanced osteoarthritic changes (Figure 1).

**Figure 2:**
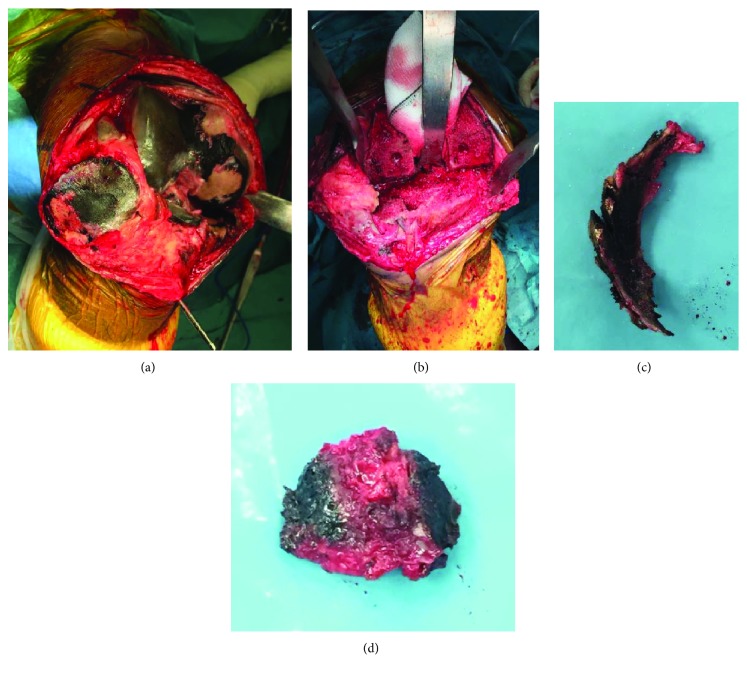
Intraoperative images of the right knee. Black discoloration is noted on the patella and trochlea (a), tibia (d), and menisci (c). The cancellous bone appears normal after preparation (b).

Having failed conservative therapy, TKA was offered and was agreed upon after informed consent. A midline incision was made, followed by a medial parapatellar approach. Upon everting the patella, it was noted that the patella tendon, menisci, cruciate ligaments, and cartilage over femur, patella, and trochlea were black in colour (Figure 2). With no signs suggestive of infection or malignancy, cemented posterior stabilised TKA (NexGen, Zimmer) was performed using iASSIST navigation. The patella has resurfaced.

Intraoperative tissue samples were sent for histological analysis which were reported to have deposition of large amorphous brown material in the above structures including the synovium, suggestive of ochronosis.

**Figure 3:**
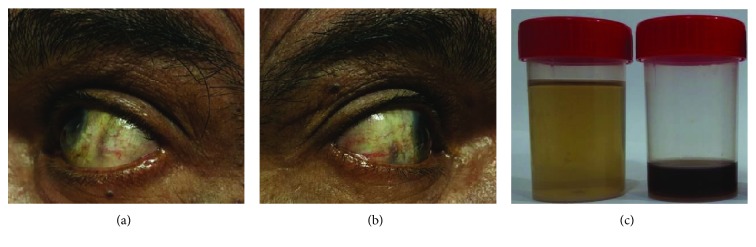
Dark pigmentation at the sclera of the left (a) and right (b) eyes. In panel (c), the normal appearance of urine on the left turned dark on the right upon addition of sodium hydroxide.

Subsequent physical examination revealed dark pigmentation of his sclerae (Figures 3(a) and 3(b)). We were unable to appreciate similar pigmentation in the ears. His urine appeared normal, until the addition of sodium hydroxide turned it dark (Figure 3(c)).

At 1 year postoperation, the symptoms in his left knee had progressed. On examination, there was medial joint line tenderness, and ROM was 15° to 90° with crepitus. Only the dorsalis pedis pulse was palpable, but the patient was advised by the vascular surgeon to manage conservatively. The patient then proceeded with left TKA.

With the same technique and implant as the right side, left TKA was performed. The operative findings were similar to the right knee. Histological analysis revealed similar findings as above.

In both instances, the patient received compression stockings, mechanical calf pumps, and 100 mg of aspirin once daily as postoperative thromboprophylaxis. He underwent the same postoperative physiotherapy protocol as other TKA patients in the institution. Continuous passive motion from 0° to 60° was started on the day of operation followed by 0° to 90° on postoperative day (POD) 1, and then 0° to maximal flexion as tolerated from POD2 onwards. Full-weight bearing was allowed immediately postoperation.

**Figure 4:**
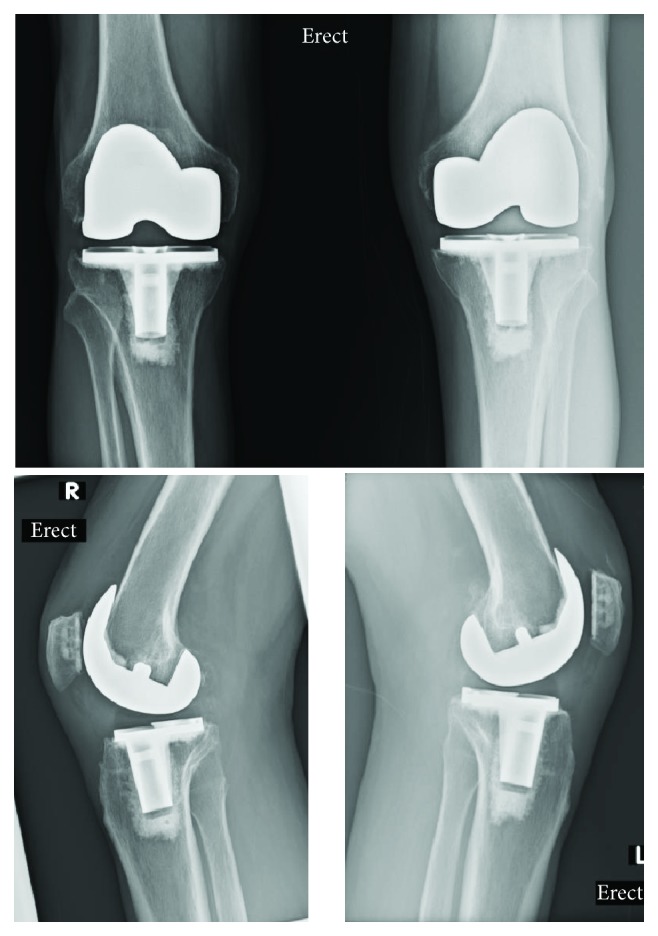
Postoperative X-rays of the knees.

At 2 years after the second TKA, he was happy with the functional outcome. He had ROM of 0° to 90° bilaterally. His Knee Society (KS) function score was 70 and KS knee score was 73 bilaterally. His Oxford Knee Score (OKS) was 40. The X-rays showed satisfactory position and alignment (Figure 4).

The patient was informed that data from the case would be submitted for publication, and gave his written consent.

## 3. Discussion

In ochronosis arthropathy, the deposition of homogentisic acid is not limited to the knees. Any collagen-rich connective tissues such as the other joints and cardiovascular system may be affected [1]. These may explain our patient's past medical history of cervical spondylosis, frozen shoulder, calcaneal tendon rupture, ischaemic stroke, and peripheral vascular disease.

To better understand the presentation and treatment success of the disease, we performed a systematic review using the Preferred Reporting Items for Systematic Reviews and Meta-Analyses (PRISMA) [12] framework as guidance. There was no existing review protocol for this study in the Database of Abstracts of Reviews of Effects (DARE) or Cochrane database or was this study registered. Databases of PubMed and Embase were searched using the terms “ochronosis knee arthroplasty”, “alkaptonuria knee arthroplasty”, “ochronotic knee arthroplasty”, “ochronosis knee replacement”, and “alkaptonuria knee replacement”. All studies containing the search terms up to October 2017 were eligible for inclusion. Titles and abstracts were screened, and full articles were obtained whenever possible using institution's access only. From the articles identified, we checked for references which were not brought up from the initial search and included them in our study. Studies were excluded if they were duplicates, non-English, or no full text available. Data extracted from the full articles included age, gender, number of joints involved, side of surgery, type of implant, when the diagnosis was made, duration of follow-up, and any indicator of preoperative and postoperative outcome, for example, range of motion, ambulatory status, complaints, or outcome scores. For the type of implant, we were interested in whether it is cemented or cement-less and cruciate substituting (CS) or posterior stabilised (PS). If no information regarding these were available from the articles, the senior author would identify the type of implants based on the radiographs provided in the relevant study. Risk of bias was assessed based on the level of evidence of the article [13]. The descriptive outcomes were regarded as the principal summary measure. Whenever possible, basic statistical data will be analysed, for example, mean age, gender distribution, and mean follow-up.

**Figure 5:**
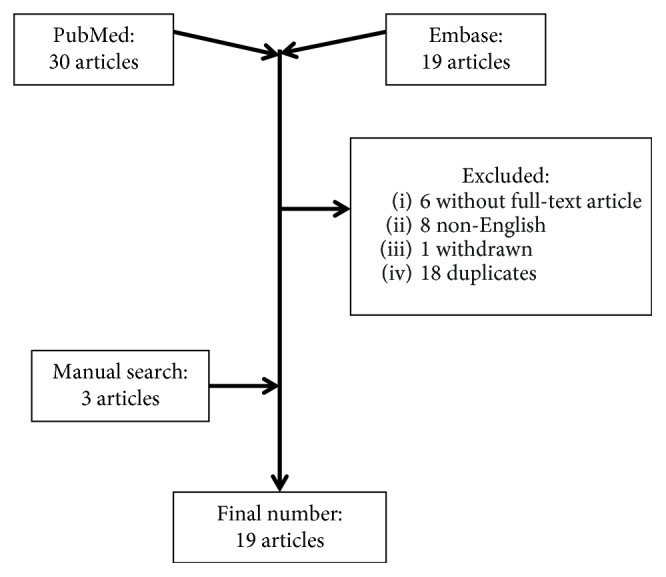
Flow chart of article selection.

A total of 19 articles between 2000 and 2016 were included in our study [2–8, 14–25] (Figure 5, Table 1). The characteristics and results of the articles are presented in Table 1. Excluding our patients, there were 19 patients comprising of 26 TKA, with the mean age of 60.5 ± 8.4 years, and 9 females (47.4%). In 10 patients (52.6%), the diagnosis of ochronosis was only confirmed postoperatively. From the data available, the average preoperative range of motion (ROM) was 91° ± 37° in 14 knees, and the postoperative ROM was 106° ± 11° in 12 knees. PS was used in 10 knees, while 5 knees had CR and 11 were unknown. The patella has resurfaced in 6 knees, retained in 9 knees, and unknown in 11 knees. Cement was used in 14 knees, not used in 3 knees, and unknown in 9 knees. From the limited outcome measures, patients appeared to have good outcome with majority claiming independent ambulatory status with minimal or no complaints. One article reported intraoperative complication of the patella tendon rupture, which was repaired with no mention of any extensor mechanism compromise in the final outcome [6]. All articles were of case reports or level IV evidences as they were part of case series [13], subjecting them to the potential of bias.

As per many other studies presented, the diagnosis of ochronosis arthropathy in our case was only made postoperatively. The finding of black connective tissues by an orthopaedic surgeon may be the first point of suspicion for alkaptonuria in the patient. Cutaneous manifestations such as dark pigmentation of the ear may not be obvious, especially in patients with pigmented skin such in our case. The black connective tissues may cause unnerving surprise to the surgeon. However, ochronosis arthropathy is a likely diagnosis and is therefore safe to proceed with TKA. The patient is likely to benefit from the procedure, given the good outcomes described in the reports presented. The next step is then to decide the type of implant that is suitable for this disease.

From our systematic review, we found most cases used cemented TKA. In a meta-analysis, cemented TKA offered better survival rate compared to cement-less TKA [26]. However, it was discussed that it may be due to cement-less TKA being used in younger and more active patients with good bone stock, for example, those reported by Aydoğdu et al. and Araki et al. [20, 22, 26]. However, ochronosis arthropathy has an element of inflammation [1], which may affect the bone quality in the long run, albeit no report of early loosening or subsidence in the reported cases that used cement-less TKAs. Nevertheless, we feel that cemented TKA should be considered for ochronosis arthropathy, as per for inflammatory arthritis such as rheumatoid arthritis [27, 28]. Furthermore, cemented TKA offers advantages such as easier technique ensuring greater primary stability, and delivery of local antibiotics [29].

In inflammatory disease, the posterior cruciate ligament may be eroded over time and cause instability in a cruciate retaining TKA [30]. This may cause failure of TKA and necessitate a revision surgery [30]. Similarly, with the potential inflammatory component of ochronosis arthropathy, a posterior substituting TKA may be the preferred option. In our case, the intraoperative assessment of the pigmented ligament was of questionable reliability and hence the decision for posterior substituting TKA.

With regards to the patella, we also prefer to resurface it, as the pathology of ochronosis involves depositing of the offending homogentisic acid in the cartilage [1]. If retained, there is a possibility of chemical irritation causing joint inflammation [1], and therefore knee pain following TKA. This is interpreted from a better pain relief and functional outcomes in rheumatoid patients who had patella resurfacing in a prospective randomized control trial [31]. However, the patella tendon may be brittle from the chemical irritation and deposition of homogentisic acid [1]. The surgeon would need to be mindful during the eversion of the patella or perform the resurfacing with only patella retraction [32].

Although there are no data from the literature regarding postoperative rehabilitation protocol, we feel that it was no different from a standard TKA patient from our limited experience. Thromboprophylaxis may be given and early physiotherapy may be attempted.

There are limitations to our systematic review; however, the findings from this study are based on a collection of case reports which carry their own bias. The number of case reports was limited, with the most recent available report being in 2016, which was 1 year from the commencement of our systematic review. Apart from ours and reports from Patel [5], Ozmanevra et al. [8], and Aydoğdu et al. [22], there are no formal outcome scoring which makes the comparison across studies difficult. Furthermore, not all information regarding the implants used was available, which limits our interpretation. Our suggested choice of implant is also based on a theoretical understanding of its pathology having an inflammatory component, hence drawing inferences using data from rheumatoid arthritis patients may overestimate the severity of ochronosis arthropathy. There remains more to be known regarding the appropriate implant for this rare condition, and it is hoped that future case reports or series will include more details regarding the surgery and outcome.

In conclusion, ochronosis arthropathy should be considered as a diagnosis when faced with black connective tissue intraoperatively. Surgeons may proceed with TKA as it provides a good long-term outcome for end-stage arthropathy. As there in an inflammatory component in ochronosis arthropathy, we suggest using cemented PS TKA with resurfacing of the patella.

## Figures and Tables

**Table 1:** Description of articles.

Author	Year	Type	No. of patients	No. of TKA	Age	Gender	Side	Cemented/cement-less	PS/CR	Patella	Follow-up	Preoperative measurement	Postoperative measurement	Known diagnosis
This study	—	R	1	2	54	Male	Bilateral	Cemented	PS	Replaced	24 months	Right ROM 5-100, left ROM 15-90. OKS: 9. KSFS: 0	OKS: 40, KSFS: 70, KSKS: 73 bilateral. ROM: 0-90 bilateral	Postop
Karaoğlu et al.	2016	R	1	1	55	Male	Left	Cemented	CR	Replaced	10 years	ROM: 0-120. No laxity	None. Full activity, no pain, satisfied with outcome. X-ray normal	Preop
Cieszyński et al.	2016	R	1	1	62	Male	Left	No mention. No X-ray	No X-ray	No mention. No X-ray	Unknown	Unknown	None	Postop
da Silva Martins Ferreira et al.	2014	R	1	2	67	Male	Bilateral	Cemented	PS	Retained	Right: 18 months. Left: 6 months	Unknown	Right knee ROM: 0-110. Left knee ROM: 0-120. Asymptomatic, walks without gait support	Preop
Patel	2015	R	1	1	58	Female	Right	Cemented	PS	Retained	18 months	Antalgic gait. ROM: 0-95	KS: 84. X-ray normal	Postop
Sahoo et al.	2014	R	1	2	51	Male	Bilateral	Cemented	PS	Replaced	28 months	ROM 10-20 bilaterally	Walking pain free. ROM 0-90 bilaterally. X-ray normal	Postop
Acar et al.	2013	R	1	1	62	Female	Left	Cemented	CR	Retained	18 months	Unknown	None. Adequate ROM and pain free. X-ray normal	Preop
Ozmanevra et al.	2013	R	1	2	69	Male	Bilateral	Cemented	PS	Replaced	2 years	BMI 30.2. Right ROM: 0-110. Left ROM: 0-114	Bilateral ROM 0-114. HSS score 95 bilaterally. X-ray normal	Postop
Reed et al.	2012	R	1	1	55	Female	Left	Cemented	No X-ray	Retained	3 months	Independent ambulation. Antalgic gait. ROM 5-120. No laxity	Excellent stability, pain relief, ROM 0-110. X-ray normal	Postop
Chang et al.	2009	R	1	1	77	Male	Left	No mention. No X-ray	No X-ray	No mention. No X-ray	Unknown	Unknown	Unknown	Postop
Kopeć et al.	2007	R	1	2	66	Female	Both	Cemented	PS	Retained	Unknown	Right ROM 0-90, left ROM 0-100	ROM 0-100 bilaterally. No pain	Preop
Kastsiuchenka et al.	2013	R	1	1	48	Female	Unknown	No mention. No X-ray	No X-ray	No mention. No X-ray	Unknown	None	None	Postop
Harun et al.	2014	R	1	1	60	Female	Left	Cemented. Vanguard biomet	No X-ray	Retained	Unknown	Independent ambulation. Antalgic gait. ROM: 10-110	None. Adequate ROM, free of pain. X-ray normal	Postop
Abimbola et al.	2011	R	1	1	48	Male	Left	Cemented. GENESIS SPC S&N	CR	Replaced	2 years	Independent ambulation. ROM: 0-130. No laxity	None. Full activities, no pain, very satisfied	Preop
Araki et al.	2009	R	1	2	56	Male	Bilateral	Cement-less	No X-ray	No mention. No X-ray	6 years	None	Returned to relatively normal activities. Walking well with crutches and without significant knee and hip symptoms	Postop
Demir	2003	R	1	2	70	Male	Bilateral	Cemented	Inadequate X-ray	No mention. X-ray inadequate	14 years	Unknown	None	Unknown
Aydoğdu et al.	2000	R	1	1	48	Male	Left	Cement-less. APS, AlloPro, Baar	PS	Replaced	4 years	Walks with cane, 10 minutes, ROM: 25-125. KSKS: 35, KSFS: 45	ROM 5-120. KSKS: 85. KSFS: 50 (limited by hip, contralateral knee, and spine). X-ray normal	Preop
Fisher and Davis	2004	R	1	2	64	Female	Bilateral	No mention. Inadequate X-ray	CR	No mention. X-ray inadequate	3 years	None	None. Able to walk without walking aids	Preop
Fontao-Fernández et al.	2010	S	1	1	71	Female	Left	No mention. No X-ray	No X-ray	No mention. No X-ray	Unknown	Unknown	None. No complications with stability in the fixation of the implants	Preop
Spencer et al.	2004	S	1	1	62	Female	Unknown	Biomet AGC. No X-ray	No X-ray	No mention. No X-ray	7 years	Unknown	None. Asymptomatic. Independently mobile. X-ray normal	Preop

Abbreviations: R = case report; S = case series; ROM = range of motion; PS = posterior stabilised; CR = cruciate retaining; OKS = Oxford Knee Score; KS = Knee Society clinical rating system; KSKS = Knee Society knee score; KSFS = Knee Society function score; HSS = hospital for special surgery.
